# Rapid Detection and Simultaneous Genotyping of *Cronobacter* spp. (formerly *Enterobacter sakazakii*) in Powdered Infant Formula Using Real-time PCR and High Resolution Melting (HRM) Analysis

**DOI:** 10.1371/journal.pone.0067082

**Published:** 2013-06-25

**Authors:** Xian-Quan Cai, Hai-Qiong Yu, Zhou-Xi Ruan, Lei-Liang Yang, Jian-Shan Bai, De-Yi Qiu, Zhi-Hua Jian, Yi-Qian Xiao, Jie-Yang Yang, Thanh Hoa Le, Xing-Quan Zhu

**Affiliations:** 1 State Key Laboratory of Veterinary Etiological Biology, Lanzhou Veterinary Research Institute, Chinese Academy of Agricultural Sciences, Lanzhou, Gansu Province, People’s Republic of China; 2 Technical Center, Zhongshan Entry-Exit Inspection and Quarantine Bureau, Zhongshan, Guangdong Province, People’s Republic of China; 3 Technical Center, Guangdong Entry-Exit Inspection and Quarantine Bureau, Guangzhou, Guangdong Province, People’s Republic of China; 4 Animal & Plant Inspection and Quarantine Technical Center, Shenzhen Entry-Exit Inspection and Quarantine Bureau, Shenzhen, Guangdong Province, People’s Republic of China; 5 Guangzhou Airport Entry-Exit Inspection and Quarantine Bureau, Guangzhou, Guangdong Province, People’s Republic of China; 6 Immunology Department, Institute of Biotechnology, Vietnam Academy of Science and Technology, Hanoi, Vietnam; University of Houston, United States of America

## Abstract

*Cronobacter* spp. is an emerging pathogen that causes meningitis, sepsis, bacteremia, and necrotizing enterocolitis in neonates and children. The present study developed an assay integrating real-time PCR and high resolution melting (HRM) analysis targeting the *OmpA* gene for the specific detection and rapid identification of *Cronobacter* spp. (formerly *Enterobacter sakazakii*) in powdered infant formula. Eleven *Cronobacter* field isolates and 25 reference strains were examined using one pair of primers, having the accuracy of 100% in reference to conventional methods. The assay was proved to be highly sensitive with a detection limit of 10^2^ CFU/ml without pre-enrichment, and highly concordant (100%) when compared with ISO-IDF 22964 in 89 actual samples. The method performed for *Cronobacter* spp. detection was less than 24 h, drastically shortened, compared to several days using standard culturing method, it is probe-free and reduces a risk of PCR carryover. Moreover, all *Cronobacter* strains examined in this study were genotyped into two species according to their HRM profiles. The established method should provide a molecular tool for direct detection and simultaneous genotyping of *Cronobacter* spp. in powdered infant formula.

## Introduction

*Cronobacter* spp., formerly named as *Enterobacter sakazakii,* is an emerging food-borne pathogen causing neonatal meningitis, sepsis and necrotizing enterocolitis and with 40–80% fatality rate to neonates, children and even in adults [1]–[4]. It has been ranked by The International Commission for Microbiological Specification for Foods as a bacterium having “severe hazard for restricted populations, life threatening or substantial chronic sequence long duration” [5]. Phylogenetic analysis of *Enterobacter sakazakii* has been studied by Lehner et al. [6] who revealed the presence of two phylogenetically distinct lineages based on the full length sequences of 16S rRNA, ie., most of the isolates and the type strain (ATCC 29544) forming one lineage, while type strain (ATCC 51329) formed the other lineage. Recently, *E. sakazakii* has been proposed to be reclassified as five genomospecies, namely: *Cronobacter. muytjensii* (type strain ATCC 51329), *C. sakazakii* (type strain ATCC 29544), *C. turicensis, C. dublinensis* and *C. malonaticus* [7], [8].

Powdered infant formulas (PIF) are common products intended for use as a food for infants due to their simulation and suitability as a complete or partial substitute for human breast milk [9]. However, increasing number of outbreaks among infants due to the consumption of PIF contaminated with harmful bacteria, first of all, *Cronobacter* spp., have been reported [1], [2], [4]. Non-sterile preparation and inappropriate handling of PIF can lead to exacerbation of *Cronobacter* infection in infants, causing illness with fatal consequence. Rapid, sensitive, simple and accurate techniques for early diagnosis of PIF contaminating pathogens, including *Cronobacter* spp., are urgently required [10], [11].

To date, various detection and diagnostic techniques for detection of *Cronobacter* spp. have been developed, including the traditional isolation and biochemical characterization directly from culture or after enrichment, or differential screening medium to identify samples potentially contaminated. However, the conventional method for the isolation and identification of *Cronobacter* spp. is time consuming and labor intensive, since it requires enrichment culture and then inoculation to selective agar followed by phenotypic identification, taking up to 7 days [12]–[14]. A number of alternative molecular methods for *Cronobacter* spp. have been investigated, including conventional PCR assay using 16S rRNA [6], *OmpA* or *rpoB* genes [15], [16], real time PCR using TaqMan and SYBR Green [3], [9], and the loop-mediated isothermal amplification [17]. However, real-time PCR requires expensive labelled probe, and LAMP may have high risk of amplicon contamination.

Many different genotyping methods have also been used to differentiate and genotype *Cronobacter* spp., which have been divided into four pulsotypes [1], and 16 fingerprint types [18], five species by PCR-RFLP [19], seven species by MLST [20], while the most authoritative classification was five species based on many biochemical and genetic studies [7], [8]. The major disadvantage of PFGE is its low stability, and the time taken (2 to 4 days). Morever, the high costs and time consuming protocols associated with sequencing for MLST have limited its use. PCR-RFLP has also been impeded by difficulties, such as the minor differences of band sizes between some species and the non-specific primers used for amplification.

Recently, high-resolution melting (HRM) analysis for fast, high-throughput post-PCR analysis of many pathogens has been developed. The HRM has been effectively used for species identification of five human hookworm species, variation scanning for differentiation, ie. between “cattle type” and “sheep type” of *Mycobacterium avium* [21] or genotyping of human hookworm [22], *Pseudomonas savastanoi* [23], noroviruses [24], *Salmonella* serovars [25], and the identification of recent and non-recent HIV infections [26]. To date, HRM has not yet been applied with real-time PCR for detection and genotyping of opportunistic foodborne pathogens *Cronobacter* spp. The real-time PCR platform with HRM supports is single-step closed tube, which can interrogate different classes of genetic polymorphisms. Moreover, it reduces turnaround time of the assay reported here to almost 1 h, eliminates the risk of contamination, and saves expense [21]–[26]. These features make it advantages for use in microbiology laboratories.

The out membrane protein A (*OmpA*), attached to host cell and persistence within macrophages, plays an important role in the brain damage [27]. It was previously reported that the *OmpA* region is suitable for the identification of *Cronobacter* spp. with higher specificity than ITS, 16S rRNA, *zpx, gluA* and *gluB* genes [28]. In the present study, we developed a real-time PCR assay coupled with HRM analysis, which was a more rapid, technically simpler detection and typing method for *Cronobacter* spp. in powdered infant formula, for the first time, targeting the *OmpA* gene.

## Materials and Methods

### Bacterial Strains

The reference strains used in this study were obtained from the American Type Culture Collection (ATCC). ATCC 51329 and ATCC 29544 were used as positive control in the detection assay and in all experimental procedures performed. Other 11 strains of *Cronobacter* spp. were isolated from the milk powder specimens in Zhongshan Entry Exit Inspection and Quarantine Bureau, Zhongshan, Guangdong Province, China during 2008 and 2010. All of the isolates were determined by Vitek 2 compact system (bioMerieux, Co., USA), and identified ultimately by the classical method ISO-IDF 22964 [12]. The 25 non-*Cronobacter* reference strains (Table 1) including *Salmonella*, *Shigella* and *Escherichia coli* were used to demonstrate the specificity of the detection assay. All bacterial cultures were maintained on Nutrient Agar plates (BD, USA).

**Table 1:** Bacterial species used in the real-time PCR and HRM analysis.

	Bacterial species	Source of strain	Result
1	*Cronobacter muytjensii*	ATCC 51329	**+**
2	*Cronobacter sakazakii*	ATCC 29544	**+**
3	*Escherichia coli*	ATCC 11775	**–**
4	*Escherichia coli*	ATCC 11229	**–**
5	*Escherchia coli*	ATCC 25922	**–**
6	*Enterobacte aerogenes*	ATCC 13048	**–**
7	*Proteus mirabilis*	ATCC 12453	**–**
8	*Proteus vulgaris*	ATCC 6380	**–**
9	*Listeria monocytogene*	ATCC 19114	**–**
10	*Enterococcus faecalis*	ATCC 29212	**–**
11	*Citrobacter freundii*	ATCC 8090	**–**
12	*Staphylococcus aureus*	ATCC 25923	**–**
13	*Klebsiella pneumoniae*	ATCC 4352	**–**
14	*Salmonella cholerae suis*	ATCC 10708	**–**
15	*Vibrio parahaemolyticus*	ATCC 17802	**–**
16	*Salmonella Enteritidis*	ATCC 13076	**–**
17	*Salmonella Typhimurium*	ATCC 13311	**–**
18	*Yersinia enterocolitica*	ATCC 27729	**–**
19	*Yersinia ruckeri*	ATCC 29473	**–**
20	*Yersinia Kristensenii*	ATCC 33639	**–**
21	*Shigella sonnei*	ATCC 25931	**–**
22	*Campylobacter jejuni*	ATCC 33291	**–**
23	*Pseudomonas aeruginosa*	ATCC 27853	**–**
24	*Pseudomonas putida*	ATCC 49128	**–**
25	*Clostridium perfringens*	ATCC 13124	**–**

### Genomic DNA Extraction

Single colony was picked up from agar plate and inoculated into 3 mL Nutrient broth (LuQiao, China) in a flask. The bacteria were cultured at 37°C for 18 h with shaking. One ml of each bacterial culture was centrifuged at 8000 *g* for 5 min, and the bacterial pastes were subjected to DNA extraction using a SK8192 DNA extraction Kit (Sangon Biotech, China). The bacterial genomic DNA samples (in TE buffer) were stored at −20°C until further use.

### Primers, Real-time PCR and HRM Analysis

A pair of primers targeting the *OmpA* gene of *Cronobacter* spp. (ie., ESOMP5- F: 5′-GGTGAAGGATTTAACCGTGAACTT-3′ and ESOMP5-R: 5′- GCGCCTCGTTATCATCCAAA-3′), was synthesized by Takara Biotechnology (Dalian, China). Real-time PCR amplification of the *OmpA* target was performed in Lightcycler 480 (Roche, USA). The total reaction volume was 20 µL, which consisted of 1× Fast-Plus EvaGreen® qPCR Master Mix (Bio-rad, USA), reaction buffer containing dNTPs, MgCl_2_, fast-activating chemically-modified hotstart enzyme, Cheetah™ *Taq*, 0.3 µM of each primer and 1 µL genomic DNA as template.

The PCR was carried out with initiation at 95°C for 60 s, then 45 cycles of denaturation 95°C for 10 s, annealing at 60°C for 10 s and extension at 72°C for 20 s. When PCR amplification was completed, HRM analysis was performed by lowering the temperature to 60°C for 5 min, followed by increasing the temperature ramping from 60°C to 95°C at 0.11°C/s, 25 acquisitions/°C. In this process, the PCR amplicons were allowed to denature and re-anneal before the high resolution melting recording changes in fluorescence with changes in temperature (dF/dT) and plotting against changes in temperature. The HRM profile was then analyzed using HRM analysis software version 2.0.1 with fluorescence (melting curve) normalization by selecting the linear region before and after the melting transition, as reported previously [24]. The auto-group function of the software was applied to generate automatic genotype group by analyzing the normalized melting curves and clustering samples into groups with similar melting profiles. Hence the difference in the shape of the “normalized melting curve” will helped cluster samples into different subgroups, and the samples can be detected and grouped in the same time [21]–[26].

### Specificity of the Detection Assay

To evaluate the specificity of the assay, 25 reference strains (Table 1) were cultured and maintained on Nutrient Agar (Oxoid, UK) plates. A single colony of pure culture was resuspended in 1 ml sterile, deionised powdered infant formula after which the genomic DNA was extracted as described above. Genomic DNA extract was used as template in the subsequent real-time PCR reactions. The specificity of the results was based upon the melting curve analysis and real time PCR amplification curve. Moreover, the identity of the amplicons was further confirmed by sequencing (Takara Biotech, China) and subsequent alignment of the obtained sequences with corresponding sequences available in GenBank.

### Internal Control

A segment of the *OmpA* gene of *Cronobacter* spp., corresponding to nucleotides 113 to 182 (GenBank Accession No. GQ845410.1), was amplified from genomic DNA using the primers ESOMP5F/ESOMP5R to generate an amplicon. The 70 bp product was ligated into the pGEM-T Easy plasmid and used to transform competent *E. coli* DH5α cells. Plasmids with the target insert (*OmpA*-containing plasmids) were confirmed by sequencing, then quantified and used as internal control for real time PCR assay.

### Detection Limit of the Assay

Artificially contaminated powdered infant formula which were tested negative for *Cronobacter* spp. by selective plating before use were prepared under double-blind conditions by directly spiking with *Cronobacter* spp. Briefly, 100 g of powdered infant formula were added to 900 mL BPW (Buffered Peptone Water) medium (Oxoid, UK). Then, two homogenates of each sample were prepared; one was confirmed to be negative for *Cronobacter* spp. according to ISO-IDF 22964 [12], and the other one was randomly inoculated with the described dilution under a double-blind condition.

The theoretical final concentration of *Cronobacter* spp. in each contaminated sample was determined using plating techniques. This was achieved by plating 200 µl of each serial dilution of *Cronobacter* spp. used on a nonselective media (Nutrient Agar, BD) in triplicate. Following overnight incubation at 36±1.0°C, the average count was determined for each dilution and the theoretical concentration of *C. sakazakii* per powdered infant formula sample was stated as CFU/ml. After artificial contamination (ranged from 10 to 10^7^ CFU/mL) with diluted *Cronobacter*, aliquots of 1 mL of homogenates were collected, then genomic DNA was extracted and real-time PCR assay was performed in triplicate.

## Results

### Specificity of the Detection Assay and Confirmation of Amplicon Identity

**Figure 1:**
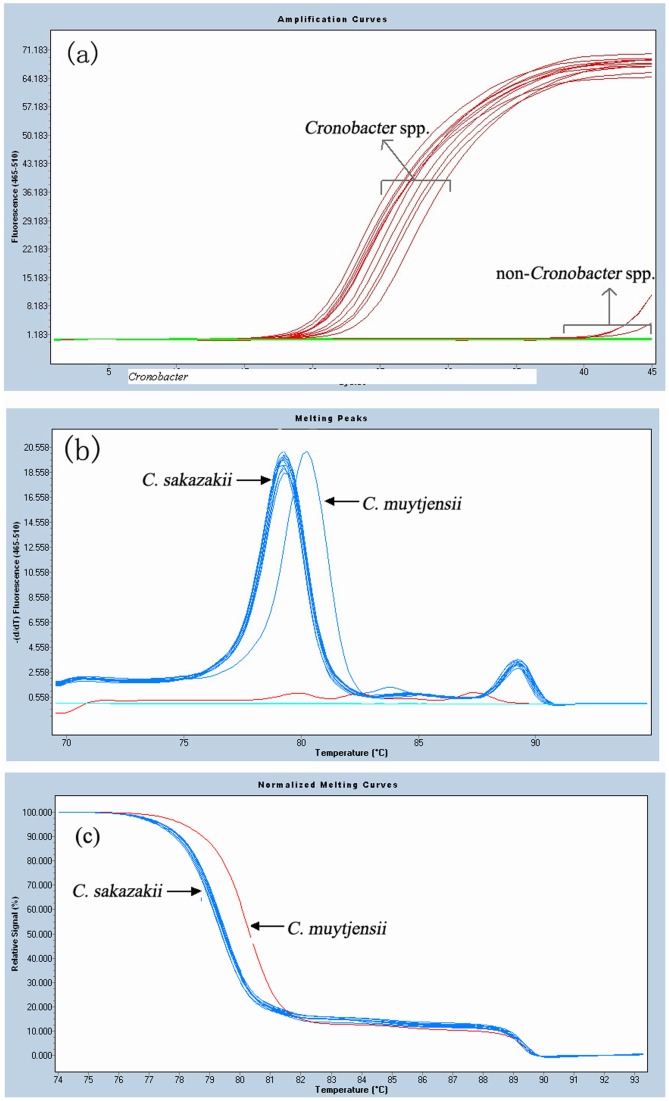
Real time PCR and HRM analysis for all bacterial strains. (a) Real time PCR amplification for *Cronobacter* spp. and other reference strains. All the non-*Cronobacter* strains and blank control had no amplification curve before 40 cycles. (b) Melting peaks of *Cronobacter* spp. 1, *C. sakazakii* including type strain ATCC 29544 and all isolates, Tm, 79.23±0.05°C. 2, *C. muytjensii* including type strain ATCC 51329, Tm, 80.11°C. (c) Normalized melting curves for *Cronobacter* spp. strains.

The specificity of the *OmpA* primers was evaluated using 11 *Cronobacter* isolates and 25 reference strains. Only strains of *Cronobacter* spp. produced positive signal (Figure 1a). All the non-*Cronobacter* strains and blank control had no amplification curve before 40 cycles. Agarose gel electrophoresis indicated that positive amplification products correlated to a size of 70 bp as expected, and nucleotide sequence analyses of the amplicons confirmed 100% identical to *C. sakazakii* (type strain ATCC 29544), while a difference of two bases was detected between *C. sakazakii* and *C. muytjensii* (type strain ATCC 51329) (BLASTn option, GenBank).

### Detection Limit and Reproducibility of the Assay

**Figure 2:**
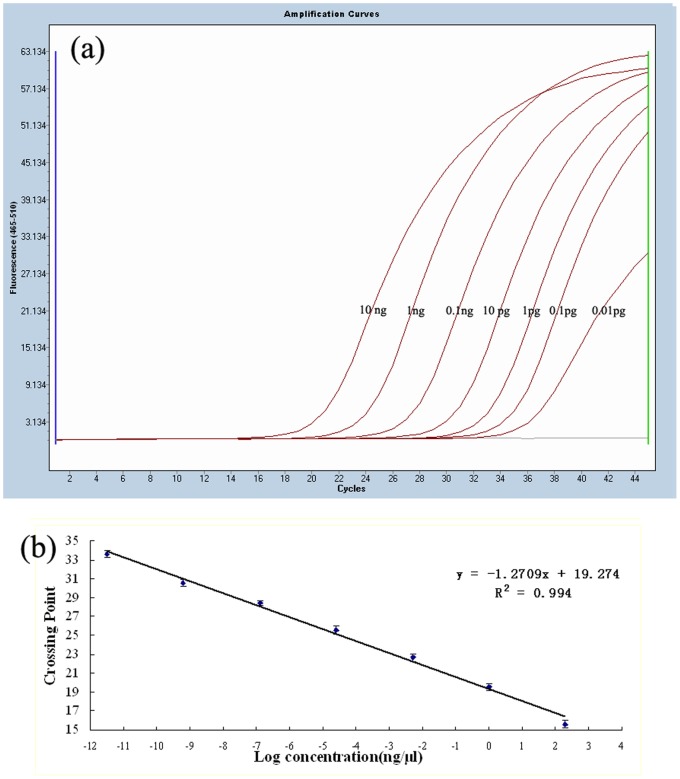
Real time PCR and linear correlation of 10-fold serial dilution of*Cronobacter* spp. genomic DNA. (a) amplification curves of diluted genomic DNA of ***Cronobacter*** spp., 1–7: dilutions 10 ng to 0.01 pg, respectively. (b) a linear regression of the data providing a formula of y = −1.2709x+19.274(R^2^ = 0.994), dilutions 10 ng to 0.01 pg, respectively.

Using powdered infant formula samples artificially contaminated with *Cronobacter* tenfold serial dilutions were performed by dissolved into BPW at 10–10^7^ CFU/mL to assay the analytical sensitivity. The results demonstrated that the lower limit of detection of samples without pre-enrichment was 10^2^ CFU/mL. Separately, a series of 10-fold diluted genomic DNA (10 ng to 0.01 pg) was chosen to evaluate the sensitivity of the detection. The results showed that limit of the assay was 0.01 pg (Figure 2a), when serial 10 dilutions were tested along with a blank control. Besides, there a good linear correlation (R^2^ = 0.994) between the log concentrations of purified DNA (10 ng to 0.01 pg) and the crossing point (Cp) value (Figure 2b). The detection assay also displayed a high degree of reproducibility. Intra-run analysis successfully detected the presence of *Cronobacter* spp. in all 10 replicates with very little variation in Cp and melting-temperature (Tm) value. Successful detection of *Cronobacter* spp. in the same milk powder sample over a period of three consecutive days also indicated high inter-run reproducibility.

### Comparison of the Method with ISO-IDF 22964 for Detecting*Cronobacter* spp

The usefulness of the real-time PCR combined with HRM assay was also compared to the method ISO-IDF 22964 in actual samples. A total of 89 milk powder samples were investigated using the cultural method and real-time PCR with HRM in parallel, between May 2012 and March 2013. Twenty-five grams test portions were analysed in duplicate for each sample after BPW-mLST enrichments as recommended [12]. The results showed that 7 samples were positive,while 82 samples was negative, 100% concordance with the gold standard method (ISO-IDF 22964).

### HRM Analysis for*Cronobacter* spp

The sequence difference between the forward and reverse primers will bring different melting temperature (Tm) values and normalized melting curves. Constant HRM profiles with distinct Tm peaks were persistently obtained for all *Cronobacter* spp. strains. As shown in Figure 1b, there were two kinds of characteristic profiles for *Cronobacter* spp. The *OmpA* amplification product from ATCC 51329 had a Tm of 80.11°C, while the amplification product from ATCC 29544 and 11 isolates collected by us had an average 79.23°C ±0.05°C (Figure 1b).

**Figure 3:**
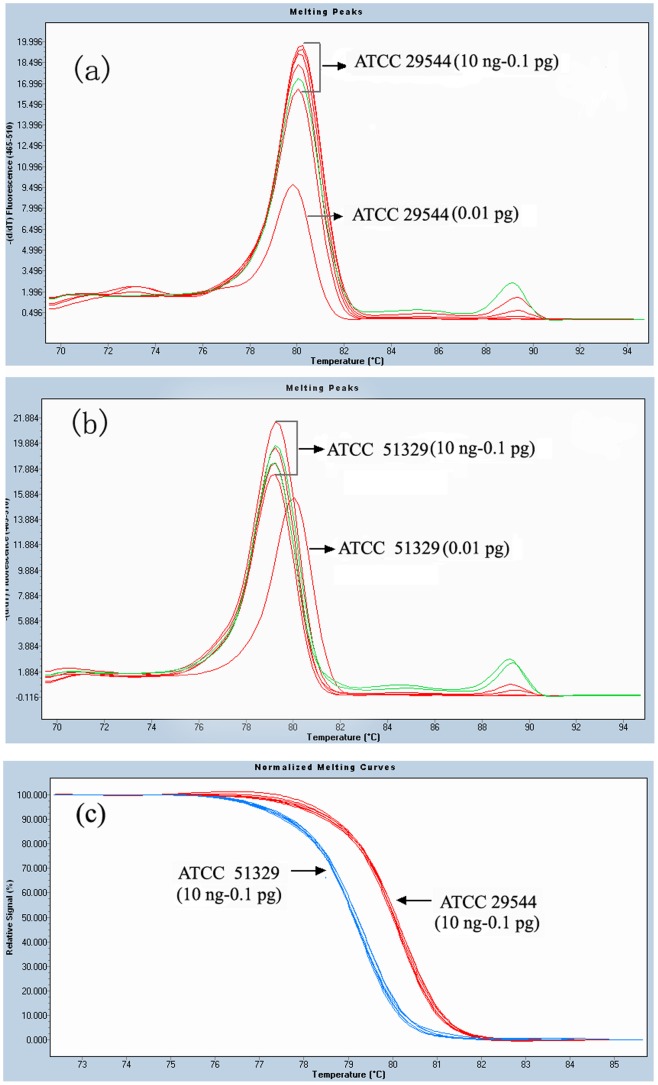
HRM analysis of 10-diluted genomic DNA (10 ng to 0.01 pg) from different type strains (a) Melting peaks of type strain (ATCC 29544), Tm, 79.21±0.04°C. (b) Melting peaks of type strain (ATCC 51329), Tm, ATCC 51329, 80.06±0.08°C. (c) Normalized melting curves of both type strains (10 ng to 0. 1 pg).

The HRM analysis with different concentrations (10 ng to 0.1 pg) of the template, appeared to be reliable. The melting peaks were obtained from each dilution (10 ng to 0.1 pg), it showed that Tm of ATCC 29544 was 79.20±0.04°C (Figure 3a), while Tm of ATCC 51329 was 80.06±0.08°C (Figure 3b). When the concentration of template was diluted to 0.01 pg, there was an obvious change, both in ATCC 51329 and ATCC 29544 (Figure 3a, 3b). Thus, the HRM profiles may be unreliable when the concentration of the template was too low (≤0.01 pg). As shown in Figure 3c, there were two obvious groups, one represented *C. sakazakii* (DNA ranged from 10 ng to 0.1 pg), and the other represented *C. muytjensii* ATCC 51329 (DNA ranged from 10 ng to 0.1 pg).

## Discussion

The currently routine procedure for detection of *Cronobacter* spp. is laborious, taking up to 7 days for completion [12]–[14]. However, even with the additional enrichment step, the method developed in this study can detect *Cronobacter* spp. in about 24 h, and reduces the need to purchase expensive probes. Real time PCR combined with HRM analysis is a new genotyping technique with advantages over PFGE, PCR-RFLP and DNA sequencing. A differentiation has also been established for genomospecies within the *Cronobacter* spp. based on sequencing after routine PCR performance [19], [20], whereas, the single nucleotide polymorphism (SNP) can be detected by HRM instead of sequencing with much less cost and time.

Real time PCR targeting ITS sequences of *Cronobacter* spp. using SYBR Green has been developed [3], however, it can only detect gross differences between amplicons generated by real-time PCR, which commonly uses non-saturating dyes such as SYBR Green which will inhibit the PCR reaction. EvaGreen has been proven to be superior over SYBR Green in general, and high reaction efficiencies can be achieved [29]. The improved instrumentation for HRM analysis with a high rate of data acquisition, ideal optics, tight temperature control and adequate analysis software has enabled us to detect single-base difference by means of HRM analysis. In contrast to traditional melting curve analysis, the information in HRM analysis is contained in the shape of the melting curve, rather than just the calculated Tm, so HRM analysis may be considered a form of spectroscopy and the accuracy of the dissociation *vs* temperature (i.e. melting) curve is as sensitive as 0.01°C [30].

We failed to find appropriate primers targeting 16S rRNA and ITS for detection and subtyping of *Cronobacter* spp. simultaneously, while acquired a good performance by using *OmpA* gene as genetic marker. Moreover, the length of PCR product is important for HRM analysis. Analyzing short amplicons can assist genotypic discrimination in HRM analysis. Whenever possible, analyzing amplicons smaller than 100 bp is preferable, especially when sites with a known polymorphism are investigated. Reducing amplicon size increases the difference in signal. Although it is highly recommended to standardize amount of DNA templates to minimize reaction-to-reaction variability in HRM assays, however, it is not found to be extraordinarily critical for distinguishing sequence difference in our study.

**Figure 4:**
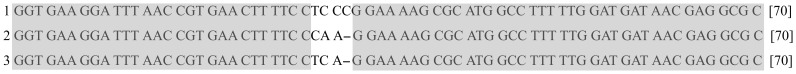
Alignment of the*OmpA* gene from *Cronobacter* spp. available in GenBank. 1: *Cronobacter muytjensii* (accession number: DQ000206.1); 2: *Cronobacter sakazakii* (accession number: GQ845410.1); 3: *Cronobacter turicensis* z3032 (accession number: FN543093.2).

It is interesting that phenotypic characteristics of all 11 *Cronobacter* isolates were consistent with that of *C. sakazakii* described previously [7]. Moreover, the *OmpA* sequences of these isolates were identical to that of *C. sakazakii.* As shown in HRM profiles in Figure 1, all 13 *Cronobacter* strains clustered into two obvious groups, one represented *C. sakazakii* (including ATCC 29544 and 11 isolates), and the other represented *C. muytjensii* (ATCC 51329). These results exhibited a clear correlation between phylogenetic analysis and HRM profiles. Currently five genomospecies are commonly recognized within the genus *Cronobacter* spp. [7], [8], but previous studies have shown that *C. sakazakii* is the predominant species, especially in PIF [19], [31]–[33]. *OmpA* sequences of three *Cronobacter* species were available in GenBank. As shown in Figure 4, there were ≥1 different bases in the segment between the forward and reverse primers in each species, which resulted in differences in melting temperature (Tm) values and HRM profiles. Therefore, the method we developed in this study has potential to differentiate all five recognized *Cronobacter* species, and this will be tested when strains representing other *Cronobacter* species become available.

In conclusion, the present study developed an assay combining real-time PCR and HRM analysis for rapid and specific detection and simultaneous genotyping of *Cronobacter* spp. in powdered infant formula. This method has potential for multi-genotying of all recognized *Cronobacter* species, and can be used for epidemiological studies of *Cronobacter* spp.
